# Telomere Attrition in Neurodegenerative Disorders

**DOI:** 10.3389/fncel.2020.00219

**Published:** 2020-07-15

**Authors:** Tina Levstek, Eva Kozjek, Vita Dolžan, Katarina Trebušak Podkrajšek

**Affiliations:** ^1^Institute of Biochemistry, Faculty of Medicine, University of Ljubljana, Ljubljana, Slovenia; ^2^Clinical Institute for Special Laboratory Diagnostics, University Children’s Hospital, University Medical Centre Ljubljana, Ljubljana, Slovenia

**Keywords:** telomere, telomere length, telomere attrition, Alzheimer’s disease, Parkinson’s disease, neurodegenerative disroders

## Abstract

Telomere attrition is increased in various disorders and is therefore a potential biomarker for diagnosis and/or prognosis of these disorders. The contribution of telomere attrition in the pathogenesis of neurodegenerative disorders is yet to be fully elucidated. We are reviewing the current knowledge regarding the telomere biology in two common neurodegenerative disorders, Alzheimer’s disease (AD), and Parkinson’s disease (PD). Furthermore, we are discussing future prospective of telomere research in these disorders. The majority of studies reported consistent evidence of the accelerated telomere attrition in AD patients, possibly in association with elevated oxidative stress levels. On the other hand in PD, various studies reported contradictory evidence regarding telomere attrition. Consequently, due to the low specificity and sensitivity, the clinical benefit of telomere length as a biomarker of neurodegenerative disease development and progression is not yet recognized. Nevertheless, longitudinal studies in large carefully selected cohorts might provide further elucidation of the complex involvement of the telomeres in the pathogenesis of neurodegenerative diseases. Telomere length maintenance is a complex process characterized by environmental, genetic, and epigenetic determinants. Thus, in addition to the selection of the study cohort, also the selection of analytical methods and types of biological samples for evaluation of the telomere attrition is of utmost importance.

## Introduction

Telomeres are tandemly repeated nucleotide sequences at the end of the linear chromosomes. Their main function is maintaining genomic stability by protecting chromosomal ends from degradation and end-to-end fusion (Blackburn and Szostak, 1984). The telomere sequence in humans consists of TTAGGG repeats, while the number of the repeats varies between different cells and even between chromosomes (Graakjaer et al., 2003). Due to the inability of DNA polymerase to fully replicate both strands of DNA at the end of linear chromosomes, telomeres become shorter with each cell division leading to replicate senescence (Shay and Wright, 2000; Martínez-Cué and Rueda, 2020). Telomere length (TL) may, therefore, serve as a biological clock indicating cellular age and is considered as a potential biomarker of aging and age-related diseases (Kong et al., 2013; Turner et al., 2019). We are reviewing current knowledge and discussing future prospective of telomere research in two common neurodegenerative disorders: Alzheimer’s disease (AD) and Parkinson’s disease (PD).

## Telomere Biology in Human and Measurement of Telomere Length

### Telomere Length Maintenance

A six-protein complex known as shelterin or telosome plays the main role in maintaining and protecting telomeres integrity and function while its dysfunction results in chromosome end fusion and DNA arrangements (Sfeir and de Lange, 2012). Components and functions of the shelterin complex are described in more detail elsewhere (De Lange, 2005). Also, the single-stranded G-rich overhang can form telomeric G-quadruplex structure (Lipps and Rhodes, 2009) that can inhibit telomere extension by blocking telomerase from accessing telomeres (Tang et al., 2008). Telomerase is a ribonucleoprotein complex that enables the maintenance of the telomere length in continuously dividing cells such as germline, embryonic, and stem cells. It consists of a reverse transcriptase subunit (telomerase reverse transcriptase, TERT) and RNA component (telomerase RNA component, TERC) that serves as a template for telomere extension (Feng et al., 1995; Harrington et al., 1997). TERC is ubiquitously expressed in all human cells while TERT expression is usually undetectable in somatic cells and increased in cancer cells (Feng et al., 1995; Meyerson et al., 1997). Telomerase function is regulated by different genetic factors and epigenetic factors associated with modulation of *TERT* transcription (Cong et al., 2002). Decreased telomerase activity or impaired telomerase recruitment leads to chromosomal instability and accelerated cellular senescence (Garcia et al., 2007; Sarek et al., 2015).

### Telomere Attrition in Health and Disease

The telomeres are longest at birth and shorten with increasing age, where the rate of telomere shortening is fastest directly after birth (Frenck et al., 1998). Telomeres in cells lacking telomerase become shorter with each cell division. When telomeres reach a certain critical length the cell exits the cell cycle and becomes senescent (Yu et al., 1990). Senescent cells secrete numerous extracellular factors such as proteases, growth factors, and inflammatory cytokines that act on non-senescent neighboring cells. Senescent cells are then eliminated by the immune system. However, this process of clearance can be impaired, especially in aged tissues, leading to the accumulation of senescent cells which contributes to further tissue dysfunction and tissue aging (Muñoz-Espín and Serrano, 2014). TL may, therefore, serve as a biological clock indicating cellular age and is considered as a potential biomarker of aging and age-related diseases (Sanders and Newman, 2013). It has been estimated that leukocyte telomeres in adult humans shorten at a rate of 24.7 bp per year (Müezzinler et al., 2013). Although, TL is synchronous within organs at the time of birth, it is highly variable in different organs of the same individual later in life (Youngren et al., 1998; Takubo et al., 2002). Besides cellular aging, several other factors can influence TL and the rate of telomeres attrition. TL appears to be longer in adult females (Benetos et al., 2001) and African Americans compared to Caucasians (Hunt et al., 2008). Furthermore, environmental factors and lifestyles such as unhealthy diet, lack of physical activity, obesity, chronic stress, smoking, and alcohol consumption have a negative impact on TL (Valdes et al., 2005; Cherkas et al., 2008; Puterman et al., 2010; Pavanello et al., 2011; Carulli et al., 2016), presumably associated with oxidative stress and inflammation (Chung et al., 2002; von Zglinicki, 2002). Due to an abundance of guanines in the telomeric repeats, telomeres are highly sensitive to damage by oxidative stress (Steenken and Jovanovic, 1997; von Zglinicki, 2002; Kino et al., 2017). Oxidative stress can arise from reactive oxygen species (ROS) or free radicals as a by-product of aerobic metabolism and ATP production in mitochondria (Reichert and Stier, 2017). Although oxidative damage can cause telomere shortening through double-stranded breaks to DNA, most telomere attrition due to oxidative stress occurs during DNA replication as a result of single-strand DNA damage (von Zglinicki, 2002). Chronic inflammation has been suggested to promote overall cell turnover leading to telomere shortening and replicative senescence (Epel, 2009; Eitan et al., 2014), therefore cumulative inflammatory load is associated with increased odds for short TL (O’Donovan et al., 2011). Moreover, inflammation can induce the release of ROS that damages telomeric DNA *via* oxidative stress (Jaiswal et al., 2000) leading to calcium release from the mitochondria, mitochondrial dysfunction, and further elevation of ROS levels (Eitan et al., 2014).

Numerous publications correlate different disorders and diseases with progressive telomere shortening. These are predominantly age-related disorders and chronic diseases such as cardiovascular diseases, hypertension, arthritis, osteoporosis, diabetes, cancer, and neurological disorders (Wentzensen et al., 2011; Kong et al., 2013; Wang et al., 2016). Nevertheless, it remains to be shown, if the observed telomere shortening is a cause for these conditions, a consequence, or both.

### Telomere Length Measurement

TL is usually measured in leukocytes considering that blood is easily accessible. Leukocytes telomere length (LTL) is considered as a surrogate marker for other cells as well. In elderly patients, TL in leukocytes is significantly shorter compared to skin, synovial tissue (Friedrich et al., 2000), skeletal muscle, and subcutaneous fat, where these tissues displayed a similar age-dependent TL attrition rate (23–26 bp/year) and the in-between tissues correlation of TL (Daniali et al., 2013). TL and attrition rates vary even among different subpopulations of leukocytes and therefore differences in leukocytes subpopulations can influence high LTL variability among individuals (Lin et al., 2015). Nevertheless, inter-individual variation in LTL distribution was stable over time (Toupance et al., 2019).

There are several methods available for measuring TL. They differ in the measured parameter (average TL or the shortest telomere), type and amount of sample required, accuracy, reproducibility, robustness, throughput, and technical skills required. Terminal Restriction Fragment (TRF) analysis using Southern blot (Harley et al., 1990) requires a relatively large amount of genomic DNA (3 μg per sample) and is labor-intensive. It is widely used because of its high reproducibility (coefficient of variation <2%; Kimura et al., 2010) and is regarded as the gold standard. Quantitative PCR (qPCR) measures the average TL in genomic DNA in a relative way, while the absolute value of average TL might be calculated using a standard curve. In comparison with TRF analysis, qPCR is easier to perform, less time consuming, requires a smaller amount of genomic DNA (50 ng per sample), and has a higher throughput (Cawthon, 2002, 2009). It is widely used for large population studies, even though there may be large variations among laboratories (Aubert et al., 2012). Pipetting errors can significantly affect the results and were partly overcome with an improved monochrome multiplex qPCR (mmqPCR) allowing the signals from the telomere amplicons and control amplicons to be collected simultaneously from the same well (Cawthon, 2009). Their main disadvantage is high intra- and inter-assay variability with reported coefficients of variation between 2 and 28% (Cawthon, 2002, 2009; Martin-Ruiz et al., 2015; Tarik et al., 2018). Single Telomere Length Analysis (STELA) is a PCR based technique combined with Southern blot that measures TL on individual chromosomes (Baird et al., 2003). It is highly accurate, requires very little starting material, and is appropriate for analysis of rare cell types. It has limited large scale applications as it has low throughput and is labor-intensive (Baird et al., 2003; Aubert et al., 2012). Telomere Shortest Length Assay (TeSLA) has similar limitations. It is a ligation and PCR-based approach for detecting amplified TRFs from all chromosomes. It detects small changes in the shortest telomeres in a certain time interval (Lai et al., 2017). Quantitative fluorescence *in situ* hybridization (q-FISH) method enables measurement of all individual telomeres per metaphase (Lansdorp et al., 1996) and can be combined with flow cytometry (flow-FISH) to measure TL in distinct cell populations (Rufer et al., 1998). Although technically more demanding, they give very reliable and reproducible results (Aubert et al., 2012).

The selection of a method should be made with consideration of their advantages and disadvantages. For larger epidemiology and population studies qPCR is the most practical choice while q-FISH and STELA are more appropriate for studies with fewer samples or when more accurate and specific measurements are required (Aubert et al., 2012). The question that remains unanswered is which parameter is more relevant for the evaluation of cellular senescence—average TL or shortest telomere? Most of the studies use methods that measure average TL rather than the shortest telomeres. While shortest telomeres are the ones that determine cellular survival and cell viability, their length might be crucial for the onset of cellular senescence (Hemann et al., 2001). Therefore, a specific group of chromosomes with the shortest telomeres might be responsible for the induction of cell senescence rather than all or only one or two critically short telomeres.

## Telomere Length in Brain

During embryonic brain development, most cells express telomerase, while after birth, the activity in most cells quickly declines to undetectable levels (Wright et al., 1996). However, high telomerase activity is present in neuronal stem cells and neuronal progenitor cells in the developing brain (Cai et al., 2002) and adult brain, particularly in the hippocampus, olfactory bulbs and subventricular zone (Martín-Rivera et al., 1998; Caporaso et al., 2003; Zhou et al., 2011) where neuronal stem cells are mainly located (Gross, 2000; Lie et al., 2004), which contribute to the viability and self-renewal potential. Telomerase activity in neuronal stem cells and neuronal progenitor cells is essential for cell proliferation, neuronal differentiation, neuronal survival, and neurogenesis (Liu et al., 2018). However, it is not sufficient to completely preserve TL in lifelong replicating cells and is leading to impaired neurogenesis and abnormal differentiation (Ferrón et al., 2009).

Studies of the relationship between TL and cognitive performance have led to inconsistent results. While TL was reported in cognitive decline in elderly people and suggested to be a possible biomarker of cognitive aging (Kljajevic, 2011; Yaffe et al., 2011; Devore et al., 2011), some studies found no significant association (Hochstrasser et al., 2012; Harris et al., 2016). Also, longer telomeres were associated with better cognitive performance (Hägg et al., 2017). The effect of short telomeres is visible in telomere biology disorders characterized by very short telomeres (Bertuch, 2016). In these patients, a central neuronal system is affected causing abnormal brain development and neuropsychiatric complications such as seizures, psychoses, mental disabilities, anxiety, and progressive memory loss (Savage and Bertuch, 2010; Bhala et al., 2019).

LTL has been proposed as a surrogate marker for the central nervous system TL (Daniali et al., 2013), yet the degree to which these measurements represent the actual state in the brain is still unclear. A *post-mortem* study in neuropsychiatric disorders reported a highly significant difference in TL in different brain regions, where telomere were the longest in *substantia nigra* and the shortest in the dorsolateral prefrontal cortex (Mamdani et al., 2015). A recent study investigated an association of short-term change in LTL and a concomitant change in structural brain plasticity. Leukocyte telomere shortening was associated with a cortical thinning, while lengthening was associated with cortical thickening, implying that short-term LTLs may be a dynamical phenomenon (Puhlmann et al., 2019).

Telomere attrition has been investigated in many neurodegenerative disorders (Eitan et al., 2014; Kota et al., 2015). In this review article, we have focused on two most common neurodegenerative disorders; Alzheimer’s and Parkinson’s disease.

### Telomere Attrition in Alzheimer’s Disease

AD is a slowly progressing neurodegenerative disease and a common cause of dementia in the elderly worldwide (Prince et al., 2016). Unfortunately, there is no effective therapeutic intervention for AD prevention (Cummings et al., 2019) while several studies have investigated possible biomarkers that would enable a diagnosis before irreversible brain damage (Aisen et al., 2017).

Amyloid-beta deposition and advanced age are the major risk factors for AD development, and both have been associated with the activation of microglia (Flanary et al., 2007) consequently leading to extensive research focusing on microglial molecules and pathways as drug targets for AD (Biber et al., 2019). A telomere shortening and reduction of telomerase activity were described in rat microglial cells during normal aging along with a tendency to telomere shortening in humans with AD dementia (Flanary et al., 2007). Amyloid deposits in dementia patients show a higher degree of microglial dystrophy compared to non-demented patients (Flanary et al., 2007). Contradictorily, studies in aged telomerase knockout mice showed that telomere shortening can decrease the progression of amyloid plaque pathology, possibly due to telomere-dependent effects on microglial activation (Rolyan et al., 2011).

Oxidative stress can trigger chronic inflammation and contribute to neuronal dysfunction and cell death in AD (Mhatrea et al., 2004; Maccioni et al., 2009) and is chronically elevated before amyloid-beta plaques and neurofibrillary tangles occur (Chou et al., 2011). Neurons expressing a high level of pathological tau were not expressing TERT. The absence of TERT led to increased damage of neurons by ROS, probably caused by hyperphosphorylated tau (Spilsbury et al., 2015).

Studies of TL in patients with AD are inconsistent. Their major observations along with the characteristics of the study cohorts and methods are summarized in Table 1. Scarabino et al., 2017, reported significantly shorter LTL in AD patients and suggested that cognitive decline is correlated with telomere attrition and conversion from mild cognitive impairment (MCI) to AD (Scarabino et al., 2017). However, stable MCI had decreased LTL compared to AD patients and controls (Movérare-Skrtic et al., 2012). Contrarily, several studies noted that TLs of the MCI patients (Zekry et al., 2010; Hochstrasser et al., 2012) or AD patients (Takata et al., 2012; Tedone et al., 2015) did not differ from the controls, but were shorter in ApoE4 homozygous AD patients (Takata et al., 2012). After the patients were classified as slow or fast progressing, the slow-progressing group unexpectedly displayed shorter TLs (Tedone et al., 2015). The increasing severity of AD was associated with a decrease in TLs and an increase in the number and aggregation of telomeres (Mathur et al., 2014). In so far the largest study with more than 300 patients followed-up for up to 16 years, deceased patients and patients who developed dementia had shorter TLs (Honig et al., 2012).

**Table 1:** Studies on telomere length in Alzheimer’s disease.

Study	Sample	TL Method	Sample size (AD/controls)	TL (AD vs. controls)
Nudelman et al. (2019)	Leukocytes	qPCR multiplex	94/223	Shorter
Flanary et al. (2007)	Microglia	Flow-FISH	4/1	Shorter
Garcia et al. (2017)	Buccal cells	3D q-FISH	44/44	Shorter
Scarabino et al. (2017)	Leukocytes	qPCR multiplex	61/56	Shorter
Liu et al. (2016)	PBMCs	qPCR multiplex	68/79	Shorter
Tedone et al. (2015)*	PBMCs	Flow cytometry	31/20	Unchanged
Kota et al. (2015)*	Leukocytes	qPCR multiplex	57/55	Shorter
Mathur et al. (2014)*	Buccal cells	3D q-FISH	41/41	Shorter
Guan et al. (2013)	Leukocytes	TRF	23/29	Unchanged
Guan et al. (2012)*	Leukocytes	TRF	40/59	Shorter
Takata et al. (2012)*	Leukocytes	qPCR multiplex	74/35	Unchanged
Movérare-Skrtic et al. (2012)	Leukocytes	qPCR monoplex	32/20	Unchanged
Hochstrasser et al. (2012)*	Monocytes	TRF	18/14	Shorter
Honig et al. (2012)*	Leukocytes	qPCR monoplex	314/1.469	Shorter
Zekry et al. (2010)	Lymphocytes	Flow cytometry	80/204	Unchanged
Lukens et al. (2009)*	Cerebellar neurons	qPCR monoplex	29/22	Unchanged
Thomas et al. (2008)*	Leukocytes	qPCR monoplex	54/26	Shorter
	Buccal cells		54/26	Shorter
	Hippocampal cells		13/9	Longer
Franco et al. (2006)*	Hippocampal neurons	q-FISH	8/7	Shorter
Honig et al. (2006)	Leukocytes	qPCR monoplex	125/132	Shorter
Panossian et al. (2003)*	PBMCs	TRF	15/15	Shorter

Abbreviations: TL, telomere length; AD, Alzheimer’s disease; q-FISH, quantitative fluorescence *in situ* hybridization; PBMCs, peripheral blood mononuclear cells; TRF, terminal restriction fragment analysis; qPCR, quantitative polymerase chain reaction. *Included in the meta-analysis (Forero et al., 2016).

Guan et al. (2012) published shorter LTLs and increased oxidative stress in AD patients of both genders, however, after vitamin E administration oxidative stress lowered (Guan et al., 2012). Interestingly, one year later they found no significant difference between women with AD and controls but a decreased number of the longest telomeres (>9.4 kb) was observed (Guan et al., 2013).

A few studies investigated TLs in brain tissue. *Lukens et al., 2009*, reported a direct correlation between TL in leukocytes and cerebellum, but cerebellum TLs were not significantly different in AD patients (Lukens et al., 2009). Shorter TLs were observed in leukocytes and buccal cells in AD patients, however, their TLs in hippocampal cells were inconsistently reported as longer (Thomas et al., 2008) or shorter (Franco et al., 2006).

The data regarding the involvement of telomere attrition in AD progression is contradictory and needs further elucidation. The common characteristic of all these studies but one (Honig et al., 2012) is a small cohort of patients (Table 1), which is probably the main reason for the inconsistency. The meta-analysis in 2016 included 13 primary studies with altogether 860 AD patients and 2,022 control patients and showed consistent evidence of shorter telomeres in patients with AD, which was more evident in leucocytes (Forero et al., 2016). A recent longitudinal study on Alzheimer’s disease Neuroimaging Initiative Cohort focused on the TL dynamics concerning late-onset AD risk and progression. Telomere attrition rate did not differ significantly between MCI and AD group, but the later did have significantly shorter TL (Nudelman et al., 2019). On the other hand, longer TL at baseline was associated with rapid progression in cerebrospinal fluid biomarkers positive patients from the same cohort (Mahoney et al., 2019).

### Telomere Attrition in Parkinson’s Disease

Parkinson’s disease is characterized by both motor and non-motor features (Kalia and Lang, 2015) where neurodegenerative cell loss is associated with chronic inflammation (Gao et al., 2003) and oxidative stress (Petrozzi et al., 2001). In PD patients, telomere shortening seemed to be related to the concentrations of carbonyl proteins in plasma (Watfa et al., 2011). It is assumed that the DNA damage leads to progressive hypomethylation in subtelomeres indicting telomeric structural changes (Maeda et al., 2012). In an animal model, telomerase deficient mice showed telomere attrition, but unexpectedly showed no differences in dopamine concentration or oxidative stress (Oeckl et al., 2014), while α-synuclein transgenic mice with shorter telomeres developed an accelerated disease with significantly decreased survival (Scheffold et al., 2016).

Studies of TLs in patients with PD showed contradictory results and are summarized in Table 2. Only two studies reported shorter telomeres in PD patients. A study in Japanese women with PD reported shorter telomeres, while length distribution showed a decrease of the long telomeres (>9.4 kb; Maeda et al., 2012). Additionally in PD, TL was reported to be shorter in buccal cells but not in LTLs (Kolyada et al., 2016). Contrary to expectations, a large case-control study in men reported shorter telomeres to be associated with a reduced risk for PD (Schürks et al., 2014). Paradoxically, longer telomeres in PBMC along with no TLs change in *substantia nigra* were also reported in PD patients (Hudson et al., 2011).

**Table 2:** Studies on telomere length in Parkinson’s disease.

Study	Sample	TL method	Sample size (PD/controls)	TL (PD vs. controls)
Kolyada et al. (2016)	Buccal cells	qPCR multiplex	30/34	Shorter
	Leukocytes			Unchanged
Degerman et al. (2014)*	Leukocytes	qPCR monoplex	136/30	Unchanged
Schürks et al. (2014)*	Leukocytes	qPCR monoplex	408/809	Longer
Maeda et al. (2012)	Leukocytes	TRF	17/20	Shorter
Hudson et al. (2011)*	PBMCs	qPCR monoplex	109/99	Longer
	*Substantia nigra*		28/17	Unchanged
Watfa et al. (2011)*	Leukocytes	TRF	20/15	Unchanged
Eerola et al. (2010)*	Leukocytes	qPCR monoplex	131/115	Unchanged
Guan et al. (2008)*	Leukocytes	TRF	28/27	Unchanged
Wang et al. (2008)*	Leukocytes	qPCR monoplex	96/172	Unchanged

Abbreviations: TL, telomere length; PD, Parkinson’s disease; PBMCs, peripheral blood mononuclear cells; TRF, terminal restriction fragment analysis; qPCR, quantitative polymerase chain reaction. *Included in the meta-analysis (Forero et al., 2016).

The majority of the studies reported no significant telomere attrition in PD (Table 2). A Swedish prospective population-based study on idiopathic parkinsonism reported no significant difference between PD patients and controls at baseline and during follow-up while longer telomeres were a risk factor for the development of dementia (Degerman et al., 2014). A study in Japanese men with PD reported no statistical difference in TLs. However, a significant decrease in TL was observed in PD patients in the 50s and 60s (Guan et al., 2008).

Reported studies evaluating TL in PD are very heterogeneous regarding the study population, sample type, TL detection method, and the cohort size. Even though telomere attrition was observed in only two studies with 30 or fewer participants and different sample types (Maeda et al., 2012; Kolyada et al., 2016), while longer TL was reported in two studies investigating significantly larger cohort (Hudson et al., 2011; Schürks et al., 2014), the contradictory observations are still surprising. The meta-analysis performed by Forero et al., 2016, included eight primary studies with altogether 956 PD patients and 1,284 controls showing no consistent evidence of telomere attrition in PD (Forero et al., 2016).

## Discussion

Studies of telomere dynamics in AD and PD have provided inconclusive results. Currently, the telomere attrition is believed to be associated with AD (Forero et al., 2016), but there is no consistent evidence of telomere attrition in PD (Forero et al., 2016). TL maintenance is a complex process characterized by environmental, genetic, and epigenetic determinants. Thus, the variability in oxidative stress and inflammation and other confounders in individuals with neurodegenerative diseases listed above may partially explain inconsistent results. Therefore, before the study, exclusion criteria must be carefully chosen to avoid conditions with increased telomere erosion. Since the telomeres progressively shorten with increasing age, different age groups should not be compared and the use of the appropriate age-matched controls is crucial.

The clinical benefit of TLs as a biomarker of neurodegenerative diseases is questionable due to its low specificity and sensitivity. Telomere shortening is not present only in neurodegenerative diseases, but also in a vast amount of other diseases and different demographic factors may contribute to telomere attrition. A recent study by *Smith et al*., using an umbrella review approach where findings were derived by examining the epidemiological credibility of the evidence, pointed out several weaknesses in reporting relationship between TL and different disorders (Smith et al., 2019). They showed no or merely weak association between TL and several health outcomes, however, the association with AD risk was suggestive (Smith et al., 2019). Nevertheless, it is still under discussion if telomere shortening is a cause or consequence in various disorders (Kordinas et al., 2016). Namely, pathological conditions may cause telomere attrition but short telomeres can also by itself induce pathological processes by increasing oxidative stress and inflammation. Therefore, it is of utmost importance that limitations of the individual study approach are considered when interpreting the data and to keep in mind that the TL as a biomarker by it selves might only give insight into the current state of the disease.

Different study designs, methods for TL measurement, and tissues in which TLs are investigated may contribute to the variability between studies. Moreover, although qPCR is the most commonly used method in TL measurement, there are still inconsistencies in recent reports. Recently, Lin et al. carefully evaluated all critical steps in qPCR telomere length assay and provided guidance and recommendations for each step based on current knowledge (Lin et al., 2019). Therefore, further efforts should be made to develop and implement standardized protocols. The experimental approach should be chosen regarding its applicability for a particular purpose of the study and specific disadvantages and advantages. External and internal controls are essential for the reproducibility and precision of the measurements. All reports evaluating TL should include the minimum information necessary to characterize the used method (Lin et al., 2019) including basic quality metrics enabling comparison of results from different laboratories.

One of the most important aspects is the selection of the representative tissue that corresponds to the pathological processes in the affected tissue, such as the brain in neurodegenerative diseases. The discrepancy in TL in different brain regions (Mamdani et al., 2015) is raising the question to what degree LTL is informative in neurodegenerative disorders. Large *post mortem* studies of TLs in different brain cells are needed to fully evaluate the LTL as the appropriate surrogate marker for the central nervous system.

The number of subjects to show statistical significance between two groups is a crucial factor and must be calculated taking into account high inter- and intra-variability in TLs. In most reported studies, the sample size was relatively small, which may be the cause for statistical insignificance. Furthermore, classification of the patients into subgroups, such as progression rate, disease stage, etc. may be beneficial to show statistical significance, but it requires an even larger total sample size. In this view, the longitudinal studies in carefully selected cohorts may provide more reliable results since a smaller sample size is needed (Aviv et al., 2006) and they enable evaluation of the telomere attrition dynamics. Such studies may assist in the clarification of the role of telomere attrition not only in development but also in the progression of the disease. That has proved beneficial in our previous studies focusing on telomere attrition in type 1 diabetes. Our initial study in pediatric patients did not confirm the association between TL and glycemic control (Tesovnik et al., 2015) contrary to the later retrospective longitudinal study (Tesovnik et al., 2018). Furthermore, integration of the clinical, genomic, and biochemical data from longitudinal studies in a multivariable predictive model may be beneficial in an attempt to identify additive biomarkers influencing the development and/or progression of AD and PD.

To conclude, longitudinal studies in carefully selected cohorts and sample types are required to elucidate the complex role the telomeres may have in the pathogenesis of neurodegenerative diseases and possible targets for intervention with a final goal to possibly prevent or delay neurodegenerative diseases. Furthermore, the integration of various experimental and clinical data might improve the strength of such studies. Additionally, standardized protocols for TL measurements addressing sample types and methods might be beneficial since and would enable a more relevant comparison of results from different laboratories.

## Author Contributions

TL and EK wrote the manuscript. VD revised the manuscript. KT conceptualized, wrote, and revised the manuscript. All authors reviewed and approved the final manuscript.

## Conflict of Interest

The authors declare that the research was conducted in the absence of any commercial or financial relationships that could be construed as a potential conflict of interest.

## Abbreviations

AD: Alzheimer’s disease
LTL: leukocytes telomere length
MCI: mild cognitive impairment
PD: Parkinson’s disease
ROS: reactive oxygen species
TL: telomere length
TRF: terminal restriction fragment.
